# Computational Convolution of SELDI Data for the Diagnosis of Alzheimer’s Disease

**DOI:** 10.3390/ht7020014

**Published:** 2018-05-17

**Authors:** Destiny E. O. Anyaiwe, Gautam B. Singh, George D. Wilson, Timothy J. Geddes

**Affiliations:** 1 Department of Mathematics and Computer Science, Lawrence Technological University, Southfield, MI 48075, USA; 2 Department of Computer Science and Engineering, Oakland University, Rochester, MI 48309, USA; singh@oakland.edu; 3 William Beaumont Hospital, Royal Oak, MI 48073, USA; George.Wilson@beaumont.edu (G.D.W.); Timothy.Geddes@beaumont.org (T.J.G.)

**Keywords:** matrix data points, mass spectra SELDI technique, Alzheimer’s disease diagnosis, matrix projection, data transformation

## Abstract

Alzheimer’s disease is rapidly becoming an endemic for people over the age of 65. A vital path towards reversing this ominous trend is the building of reliable diagnostic devices for definite and early diagnoses in lieu of the longitudinal, usually inconclusive and non-generalize-able methods currently in use. In this article, we present a survey of methods for mining pools of mass spectrometer saliva data in relation to diagnosing Alzheimer’s disease. The computational methods provides new approaches for appropriately gleaning latent information from mass spectra data. They improve traditional machine learning algorithms and are most fit for handling matrix data points including solving problems beyond protein identifications and biomarker discovery.

## 1. Introduction

Alzheimer’s disease (AD) is rapidly becoming endemic to people over the age of 65. In most patients, neuro-degeneration begun many years prior to the clinical diagnosis of the disease. Unfortunately, there are no clinical tools to detect the early stages of the disease. Patients and clinicians are hampered by the lack of definitive diagnoses, since it usually take several years of surveillance before the disease is recognized. There are no cures for the disease, but if early diagnostic techniques were to be available, there are available medications that can alleviate the neurological symptoms and degradation.

Currently, the diagnosis of AD is usually made when the patient has undergone irreversible neurological damage. Therefore, current drug treatments are ineffective. Thus new methods to diagnose the disease are urgently required.

Objects and observations in research are often represented and implemented as vectors. Coupled to this, machine learning algorithms are designed to function with vector data points as inputs and outputs. This becomes problematic upon the need to to mine other types of data structures. The aim of this report is to extend machine learning algorithms towards accommodating matrix data points. A typical source of these type of data points are high-throughput assay such as mass spectrometry (MS).

Mass spectrometry is an assay technique used for the analyses of biochemical specimens. its paradigm entails specimen ionization, acceleration, deflection and detection of ion species and usually yields tens/hundreds of ions sorted based on their mass-to-charge ratio. Differentially expressed ions are of key importance in MS results, they are targeted, identified, and isolated for further investigation.

The literature contains reports of advances made using results of different mass spectrometer analysis techniques in protein identification and biomarker discovery. Papers like ([1] and [2]) discus disease diagnosis but not much progress has been registered for the subject under review. To our knowledge, there are no methods for instant, accurate and definite AD diagnoses.

The unique reason behind the idea of using saliva as protein source sample (PSS) is discussed in this report. The methodologies presented develop algorithms for the convolution of matrix data points. The possibility of successfully mining mass spectrometer saliva data increases the goal of developing diagnoses tools for Alzheimer’s diseases.

The data used in the reports under review is as a result of surface-enhanced laser desorption/ionization (SELDI) time-of-flight technique. Although, this is an old technique the potency in the data it generates is fully established in this report via the results obtained (and presented). The list of common limitations associated with MS-SELDI proteomics acquisition techniques includes questions concerning the accuracy in detecting valid regions for ion selection, reproducibility of candidate ions, reliable identification & quantification processes, and ambiguity problems.

Generally, for detectable regions and reproducibility, proteomics is concerned with ensuring that every PSS analyzed is completely ionized in a given chromatographic time, and that, such level of ionization is repeated in similar assay processes. John et al. in [3], presented a multiplexed and data-independent acquisition (DIA) technique. Data-independent acquisition is aimed at guaranteeing that the regions for data acquisition is dynamic, and not restricted to only the best ionized regions. It showcased steps for examining ‘multiple’ precursor ions in parallel as against the ‘one peak at a time’ examination. Akin to multiplexed acquisition is SEQUEST ([4,5]) which matches spectra ions to peptide sequences based on cross-correlation comparison of observed spectrum against theoretical spectrum.

Mass spectrometry-SELDI data peak identification and intensity quantification problems still arise for both ‘*one peak at a time*’ and ‘*multiple peaks*’ examination scenarios. In such instances, proteomics is concerned with defining the standard criteria to be adopted for identifying and quantifying candidate precursor ions, including what information should be gleaned from them.

Another issue is that it is usual to encounter several proteins that share similar precursor or peptide ions. In these regards, several models such as probability based search procedures have been proposed to tackle this abnormality, including Mascot search engines [6], and target decoy approach ([7,8]). PeptideProphet is another model that has been developed, which statistically validates observed spectrum against a theoretical spectrum [9,10]. A comprehensive comparison of these pre-processing algorithms for SELDI data has been published [11]. The study established that different conclusions are obtained for different experiments based on the tuning of the involved parameters (laser bombardment energy level, proteinChip, choice of algorithm, pre-processing steps, etc.).

There are attempts at providing unified MS data repositories; ProteomicsDB [12], ProteinXchange [13] and the human proteome project (HPP). It will take time and consideration of several issues before a unified platform can be created. In particular, before mining any publicly available data for diagnosing AD, the info about the health (neurological) status of the PSS used in the MS analysis of the dataset has to be known.

In this light, we propose computational methods for the convolution of matrix data points or Mass Spectra data are in general. These methods are algorithmic improvements of machine learning algorithms, designed as tools in seeking possibilities of doing more with MS-SELDI (high-throughput) data against the odds of experimental origin, ill-posedness and structure. In this study, we focus on designing highly interactive disease classification (diagnoses) mechanisms using AD as a case study.

This report establishes that conventional machine learning algorithms are not admissible owing to the matrix structure of the proposed MS-SELDI data points. A review of their computational frameworks, limitations, and the future of these algorithms in biomedical fields in relation to high1-throughput assay processes in adoption of the steps introduced in these reports [14,15,16,17].

## 2. Data and Problem Formulation

**Figure 1:**
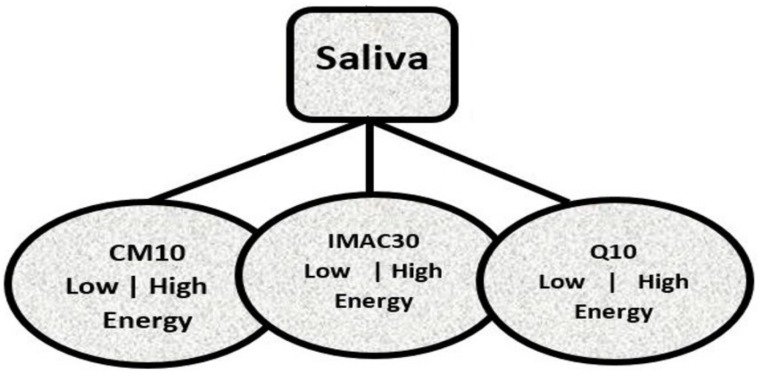
A tree representation of analysis of saliva protein source samples using three proteinChips under two levels of laser energy bombardments. See Table 1 of [18] for detailed description of CM10, IMAC30 and Q10 proteinChip arrays.

A classical problem was drawn for an MS–SELDI-TOF dataset used for the research works under review using the data tree on Figure 1. The PSS were urine, *saliva* and serum (emphasis on saliva since it was used in the reports presented here). Each of the samples were analyzed using three proteinChips; CM10, IMAC30 and Q10 under two laser bombardment energy levels; low (1800 nj) and high (4000 nj). Where each leaf of Figure 1 establishes a cohort of the 3 stages of Alzheimer’s disease; CON, MCI and TAD. There were 20 MS results in each leaf defined by a PSS, a disease stage, the proteinChip used and the energy level the experiment was carried out in.

CON refers to the control group i.e, aged respondents with no case of AD. Respondents with mild cognitive impairment made up the MCI stage/group while respondent with total or acute impairments belonged to the TAD class.

Working from excel sheets, each MS-SELDI assay result is an *n-row-by-2-column* data. Where each row is a differentially expressed peak (or ion). We considered each peak as a row vector having m/z (*m*) and TOFIntensity (*I*) entries. Such that, every assay result is a *matrix*, a collection of tens/hundreds of ion species. For instance, $p_{i}^{C}=\left(m_{i},I_{i}\right)$ is a peak $p_{i}$ found in a matrix under the CON (*C*) stage.

(1) $$P_{1}^{C}=\left[\begin{array}{cc} m_{1}^{1} & I_{1}^{1}\\ m_{2}^{1} & I_{2}^{1}\\ \vdots & \vdots \\ m_{n}^{1} & I_{n}^{1} \end{array}\right]\;P_{k}^{M}=\left[\begin{array}{cc} m_{1}^{k} & I_{1}^{k}\\ m_{2}^{k} & I_{2}^{k}\\ \vdots & \vdots \\ m_{n}^{k} & I_{n}^{k} \end{array}\right].$$

**Figure 2:**
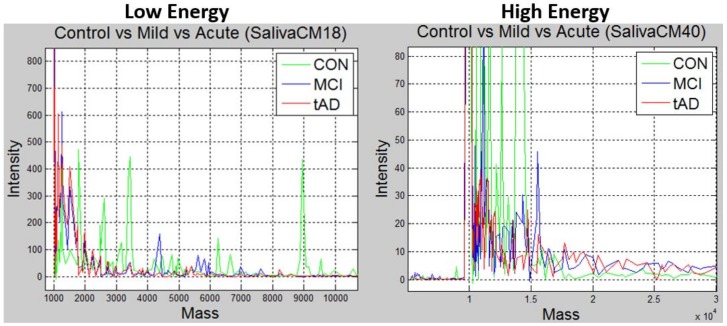
Spectrograph of unique peaks with respect to laser energy bombardment levels and disease stage. Green spectrograph are unique peaks in control stage, while blue and red spectrographs respectively represents unique peaks in mild and acute stages.

The superscripts (C,M, or *T*) and subscript (*k*) of matrix **P** denotes the stage and the result/matrix index respectively. For a peak *p* (lower cased), the *n* as in $p_{i}=\left(m_{n}^{k},I_{n}^{k}\right)$ denotes the row number the peak is occupying in *P*. The size of *n* (the number of differentially expressed peaks) differs with respect to the energy level and proteinChip used in the MS experiment. For instance, the number of differentially expressed peaks with CM10-low-energy data is 178 while CM10-high-energy produced 299 differentially expressed peaks. Henceforth, let’s call the matrix **P** a data point. Pools of ions in each cohort or leaf of Figure 1 were created, and Matlab was used to normalize them with respect to their disease stages to obtain unique peaks, Figure 2 is a snapshot of two plots based on the energy levels the experiment was carried out in. The plots are 3-in-1 plot of unique peaks in each stage for saliva CM10 proteinChip. The spectrographs on the left are from CM10-low-energy MS data, the green spectrograph represents the *CON* elements, blue for *MCI* and red for *TAD*. A similar plot is on the right for CM10-high-energy data.

Notice that there are no definite pattern depicted by the plots in Figure 2, that could be used to separate the spectrum into different stages. The research problem under review is the ability to define a separation model, which subsequent data points can be correctly classified into any of the 3 disease stages. In other words, what method can be adopted to identify *N* number of peaks in any given spectrum in e.g., Figure 2, such that subsequent spectrum of like stage/label aligns uniquely to its kind while spectrum from different stages are distinctively differentiable.

Clearly, none of the search, identification and quantification algorithms mentioned above is capable of solving this new problem. Target decoy or the matching technique of observed ions with theoretical or public database ions exhibits only the chemical properties of the quantified ion with no information about the root source of such ion. Patterns cannot be generated with them and in addition, the probability of occurrence of each of the *n* observed ions is equal for each analyzed specimen under the same experimental condition irrespective of disease stage. Thus, statistical validations also fail.

## 3. Machine Learning

A wide range of clustering/classification techniques are thought and practiced in different fields interested in data science, machine learning, knowledge discovery or information retrieval. A summary of clustering techniques in Bioinformatics was presented by Ali & Khan in [19]. As mentioned above, none of these techniques are admissible with mass spectra datasets if considered as matrices. Another classical approach of determining and indicating the similarity/difference between objects is through the use of dendrograms. This method uses appropriate choice of distance(dis-similarity) functions and objects are represented as vectors (e.g. objects in a two-dimensional plane).

### 3.1. Similarity/Distance Functions-Dendrograms

Consider only the intensity values of each data point i.e., reduce each data point $P_{i}^{S}$ described in Equation (1) to vectors $P_{i}^{S}=\left(I_{i}^{k},I_{i+1}^{k},\cdots I_{n}^{k}\right)$ and construct a dendrogram with the data. On a side note, this guarantees that the comparison is made between intensity values after they have been sorted in an ascending/descending order with reference to their mass values. Such that the obtained sequences (if consistent) could be used for classification purpose. For instance, assume that $s_{1},s_{2}$ and $s_{3}$ are the sequences of conjoined nodes for each stage based on their distances apart.

(2) $$\begin{array}{c} s_{1}=I_{i}-I_{g}-I_{h}-I_{a}-\cdots \\ s_{2}=I_{b}-I_{l}-I_{y}-I_{c}-\cdots \\ s_{3}=I_{d}-I_{j}-I_{q}-I_{s}-\cdots \end{array}$$

The generalization is hinged on the consistency and uniqueness of intensity values (in this case, nodes) that become joined together based on their pairwise distance. However, the following concerns has to be investigated

- Uniqueness: Will the sequence nodes still find and link each other in a larger and heterogeneous pool of vectors?
- Experimental factors: In the face of non-availability of universal repository/data bank, it has been established that experimental factors such as capability of mass spectrometery machines, adopted mass spectrometry technique, laboratory procedures and expertise governs the accuracy/reliability of assay results. If the $I_{i}$s happens to be consistent, what are the chances that they will (or not) be present in subsequent MS results if carried out for the same PSS.

As an illustration, a 70–30% partitioning of the dataset was made where the 30% of 20 data points in each stage made up its test data points, while the rest 70% made up the train dataset. Using Frobenius distance function (reason for this will be explained later), we determined the pairwise distances between all data points across and within each stage. Sequel to this we studied the nature of the ‘incident’ and ‘reflection’ angles of the test data points across the train data plane. The assumption is that giving objects of a stage dataset as test elements that their traversing of the plane of the train data points should be somehow consistent with respect to a set of the test data points. Thereby, developing a pattern by extension.

**Figure 3:**
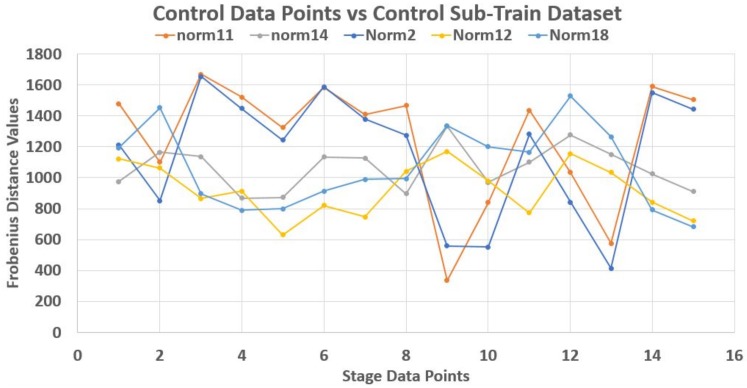
The traversing of control test data points on control train data plane.

**Figure 4:**
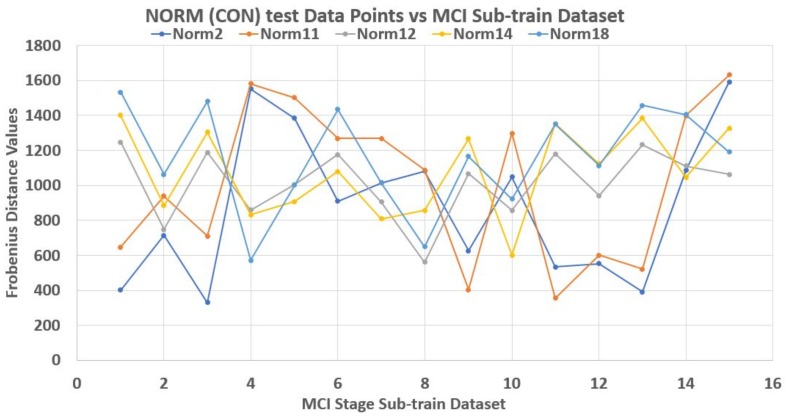
The traversing of mild test data points on control train data plane.

**Figure 5:**
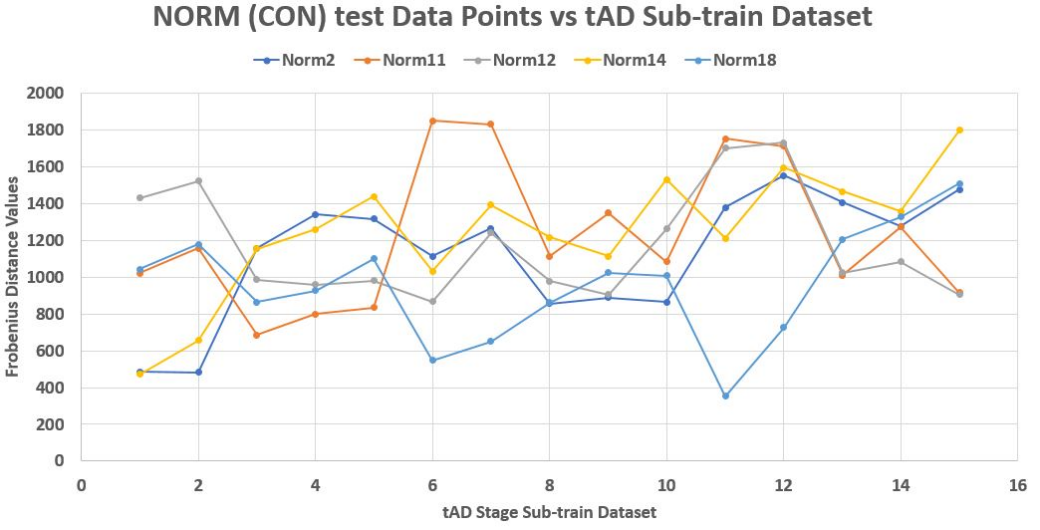
The traversing of tAD test data points on control train data plane.

Unfortunately, this is not the case. Each test element had its peculiar incident and reflection curve as seen in Figure 3, Figure 4 and Figure 5. This confirms the non-uniqueness and inconsistency of the sequences in Equation (2). Thus non generalize-able. In this figures, the test data points are from control (norm) stage. Also, Frobenius distance function was used to avoid feature selection and sorting steps since this mechanism is designed for matrix data points in general.

### 3.2. Classification by Transformation

This is the first classification model we are proposing. It uses the unique characteristics of eigen vectors and eigenvalues. The methodology entails computing or solving for the eigen vectors and eigenvalues associated with a pool of data points of a leaf or the 3 disease stages and subsequently rank the eigen vectors based on the absolute magnitude of associated eigenvalues. The outcome of this operation enabled the identification and selection of eigen vectors that represents the region or area of largest variance. The next stage is choosing the largest *N* eigen vectors.

The first two eigen vectors were chosen and concatenated to get a new algebraic property called eigenmatrix M. The size of the eigenmatrix (*n*-rows-by-2 columns) in this case, was chosen to satisfy the law of matrix multiplication.

(3) $$projection=P^{\prime }✱eigenMatrix.$$

**Figure 6:**
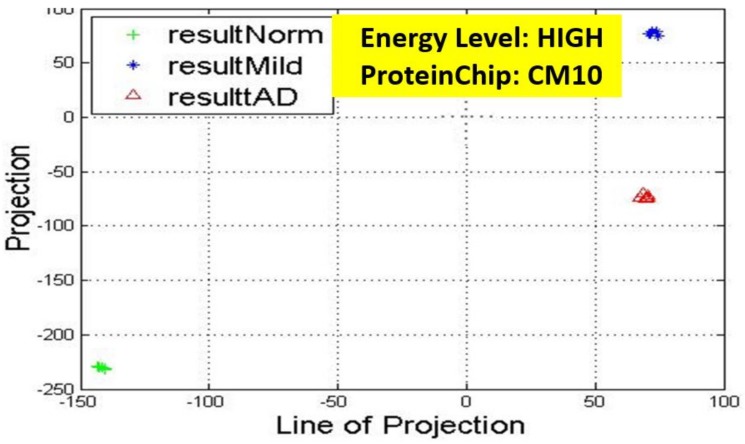
Projection results of data points by their stage eigenMatrix. The projection locations produced clusters for CM10 high energy data points. AD: Alzheimer’s disease.

**Figure 7:**
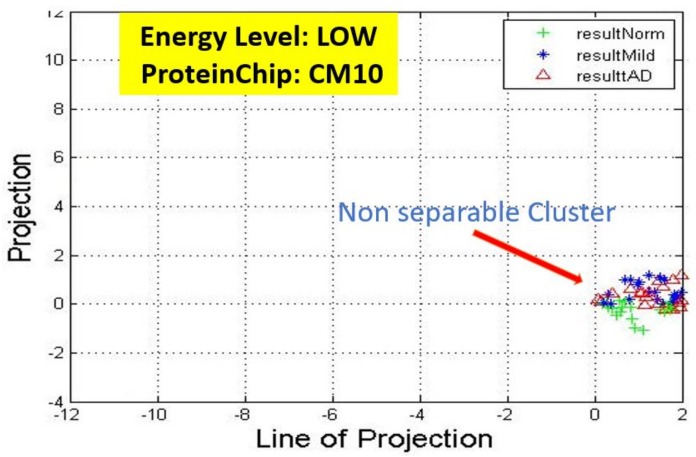
Projection results of data points by their stage eigenMatrix. For CM10 low energy data points, a scattered projections was obtained.

**Figure 8:**
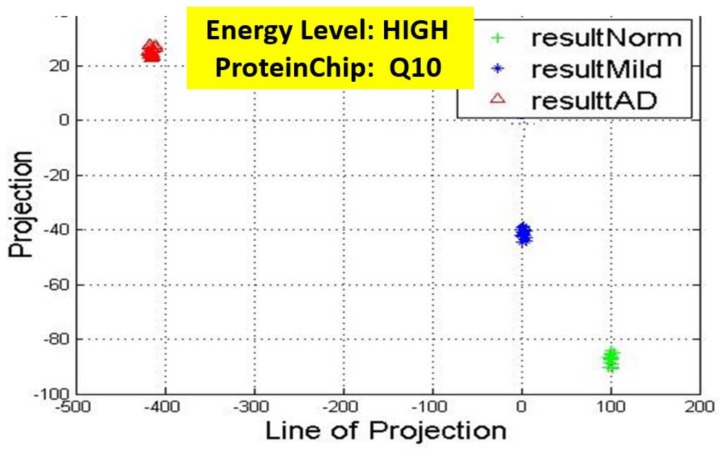
Projection results of data points by their stage eigenMatrix. The projection locations produced clusters for Q10 high energy data points.

**Figure 9:**
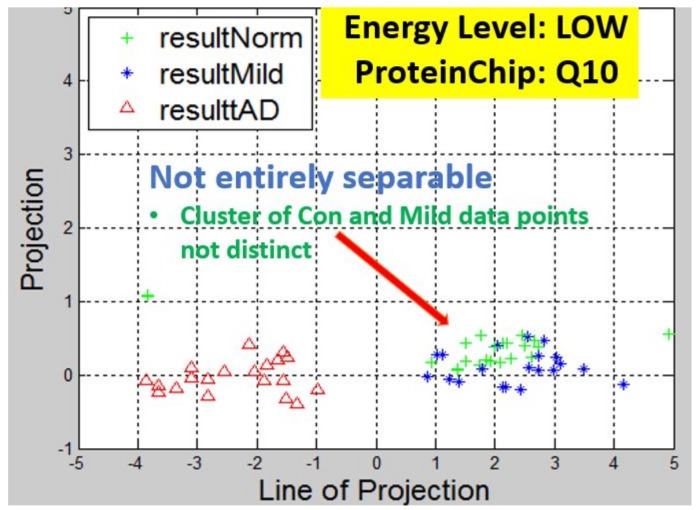
Projection results of data points by their stage eigenMatrix. For Q10 low energy data points, a scattered projections was obtained.

**Figure 10:**
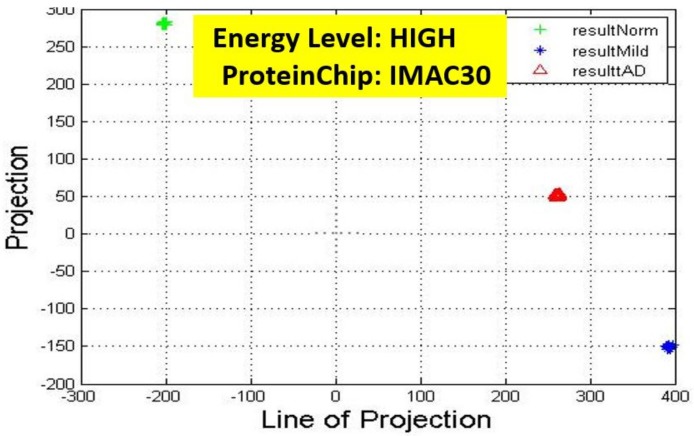
Projection results of data points by their stage eigenMatrix. The projection locations produced clusters for IMAC30 high energy data points.

**Figure 11:**
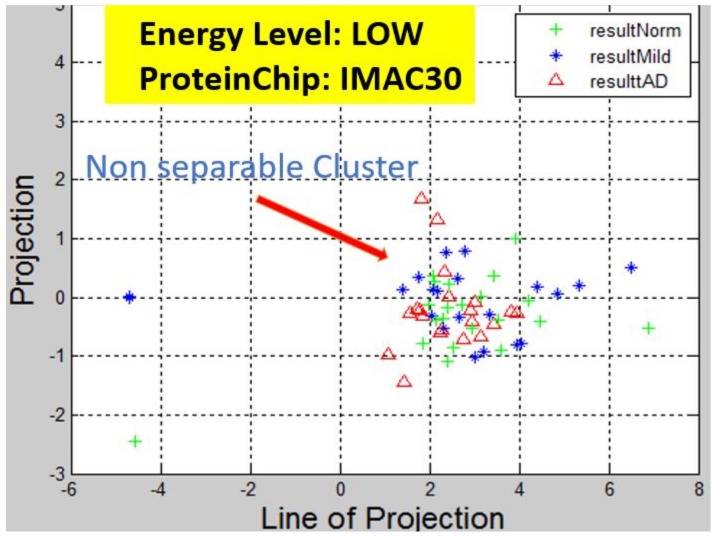
Projection results of data points by their stage eigenMatrix. For IMAC30 low energy data points, a scattered projections was obtained.

Recall, the size of P is also n−by−2. A linear transformation of M by the transpose $P^{\prime }$ of each data point (with regards to disease stage) onto a two-dimensional space was then carried out. Some outcomes of this transformations is presented in Figure 6, Figure 7, Figure 8, Figure 9, Figure 10 and Figure 11; see [16] and [17] for more details. In these figures, eigenmatrix was constructed for both low and high energy levels using each stage data points under the proteinChips used.

Observation: We begin with a definition of a *cluster*. A *cluster* is a group of similar objects occurring closely together. By this definition, researchers impose some relationship upon the closely knit objects for generalization purposes. Now, it can easily be identified that well defined and *separable* clusters were obtained for the projections of high energy data points. Secondly, cluster sizes differ (shrinks) with respect to the eigenmatrix used for the projection, i.e, robust clusters are obtained if the stage of the projected data points is the same with that of the eigenmatrix. The degree of cluster shrinkage is also proportional to disease severity.

**Figure 12:**
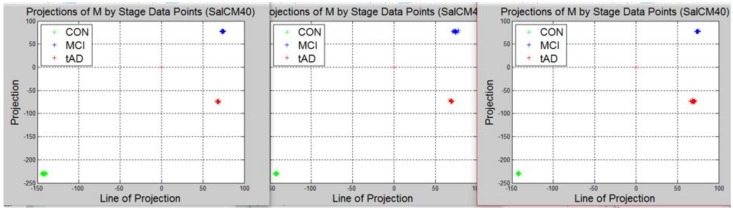
A grid view of projection results using those of CM10 high energy data points.

An immediate concern would be to determine if our observations above is unique and consistent or purely random. From Figure 7, we see a case where the projections did not generate defined clusters for the data points per their stage. In addition, consider the plot of Figure 12 which are the projections of the data points of leaf CM10-high energy.

**Figure 13:**
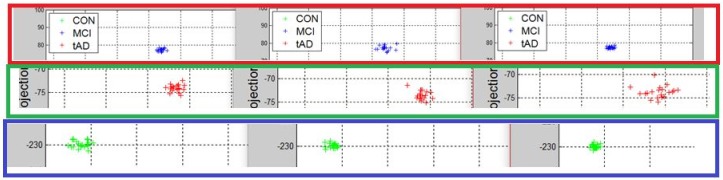
A zoomed in view of Figure 12, illustrating the structure and difference of the clusters.

Equation (4) is the grid made up of how data points and eigenmatrices were projected to yield the clusters of Figure 12. The first alphabet in each pair of alphabet corresponds to the stage label of the data used for constructing the eigenmatrix while the second alphabet indicate the stage label of the data projected the eigenmatrix. Zooming in with the same magnitude on the clusters of Figure 12 provided the plots of Figure 13.

Interpretation: To interpret this, one can either use the characteristics of the rows or columns of Equation (4). For instance, consider the projections of each data point using CON-eigenmatrix (green clusters) across the three plots and notice that *tAD* projection has the most shrunk cluster, followed by *MCI* and with *CON* cluster being most robust. In this light and given a test data, the test data can reliably be classified. The outcome of this methodology highlights the behavior of a set of data points with respect to the domain of each eigenmatrix. We identify that in general, clusters generated with eigenmatrix whose stage is same with the stage of the data point are more robust. Thus, there are regions/areas uniquely covered by such clusters when compared to their counterparts.

(4) $$\left|\begin{array}{c} MC\;MM\;MT\\ TC\;\;TM\;\;TT\\ CC\;\;CM\;\;CT \end{array}\right|.$$

### 3.3. Distance-Based Classifications

Earlier on, we saw that the dendrogram approach failed. The hypothesis that the distance between peaks (ions) of data points within a stage is less compared to those across the stages was critically explored further by [14,15].

This classification models extends the principle of determining the pairwise distance between pairs of observations. They were formulated in a manner that allows every peak vector of a matrix to play a role in its classification. In [14], the authors engaged the principle of k-nearest neighbor algorithm with a modification of the Euclidean distance function as the underlining distance function and called it exponential Euclidean k-nearest neighbor algorithm (EE-kNN). In the regular Euclidean function the square root of the sum of the squares of the terms $\Delta m=m_{a}-m_{b}$ and $\Delta I=I_{a}-I_{b}$ is computed as the distance between two vectors $v_{a}$ and $v_{b}$.

The ‘Exponential Euclidean’ distance function, computes the exponential difference between the mass inputs $m_{a}$ and $m_{b}$ using $\Delta m={\left(e^{{\left(m_{a}-m_{b}\right)}^{2}}-1\right)}^{2}$ while ΔI was computed linearly as in Euclidean distance formula. The essence of this according to [14], is to exclude vectors with large Δm since KNN seeks minimum distance for inference. An iteration entails determining the distance between each vector of a test data point (*test vector*) and corresponding vectors in train data points having equal mass values. Using the principle of KNN, a test vector is classified based on the stage that has the highest k-minimum (nearest) neighbor to it. Overall, data points are classified to the stage most represented in the classification table of its vectors.

Next is Manhattan distance classifier (MDC), [15]. This method quantifies the significance train data vectors are to test vectors using Manhattan distance formula. Test vectors are classified to the stage of the most significant vector and the test data points are classified based on the stage having the highest number of most significant vectors to its vectors. The classification results obtained with both models are presented by Table 1 and Table 2.

A sensitivity analysis that corrected errors associated with distance based classification models including the ability to predict with them was presented by [16]. In Statistics and by extension the medical practice, the power with which a methodology or a list of observations can lead to rejecting a (null) hypothesis for some defined alternative is of paramount interest. The ability to define this ’power’ can only be determined through knowing the probability of committing type II error, thus, researchers usually define a two class or YES/NO system.

Assume that diagnosing AD is a two class/stage problem for the moment, where control data points constitute the *non-disposed* class and MCI and TAD data points made up the *predisposed* class. In addition, let us define a *default* diagnosis as the diagnosis done by a seem like flipping of a coin exercise. The end point is to make a classical comparison of the probability of committing type II error via default diagnoses to that of CM10-Low-energy classification result under EE-kNN model, Table 3 and Table 4 (note that CM10-Low-energy is the poorest of all the classification results obtained).

Table 3 presents a more detailed classification result of EE-kNN-CM10-low. Since there are 20 data points in each stage and MCI & TAD has been merged to form the predisposed class, the predisposed class now has 40 data points. Same statistics was maintained for the corresponding class in default diagnosis. Type II error elements are the mis-classified data points in the cells colored red.

In numbers, committing Type II error for default diagnoses is 0.33 with a power of 67% while that of EE-kNN-CM10-Low is 0.13 with a test power of 87%. A 20% improvement! Let’s reemphasize that the EE-kNN-CM10-classification result used in this final analysis is the worst classification result obtained under distance based classification and it still performs 20% better than the default diagnoses. Although, this analogy is purely theoretical it however, pinpoints the credibility of distance based classifications presented in [14], and [15] over the usual non-scientific, usually inconclusive longitudinal follow-up system currently in use for diagnosing AD.

In [16], the authors presented a sensitive analysis of these classification results as it applies to diagnosing Alzheimer’s disease via MS-SELDI saliva data analysis when the diagnosis problem is viewed as a two-way problem. They observed that a degree of bias is introduced if the conventional two class scenario described above is adopted for evaluating the error types or test power of algorithms when it applies to three-case problems such as AD diagnoses.

**Figure 14:**
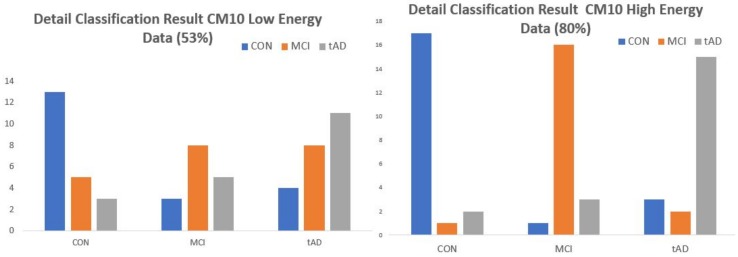
Bar chart representation of detail classification results of EE-kNN model for CM10-High and CM10-Low energy data points.

**Figure 15:**
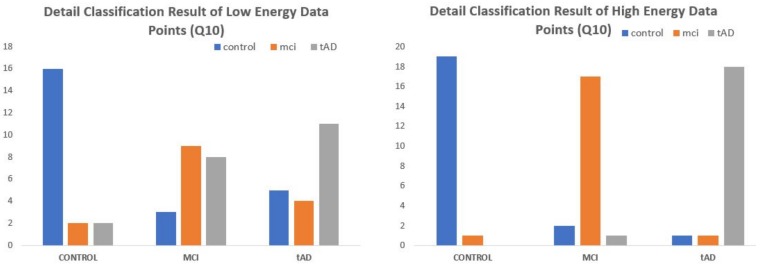
Bar chart representation of detail classification results of MDC model Q10-High and Q10-Low energy data points.

Consider the bar charts on Figure 14 for EE-kNN-CM10 low & High energy data points and Figure 15 for MDC-Q10 low and high energy data points. For each set of cohorts, blue bars represents the correctly classified CON data points under CON bracket, orange and grey are respectively the correctly classified MCI and TAD data points under MCI and TAD cohorts. Every other color/bar in each subsection represents objects that were misclassified. Under the two-class paradigm the orange and grey bars in MCI and TAD subsections would have been termed correct classifications for predisposed class leaving out only the blue bars as misclassified. Similarly, in the CON subsection, the elements of orange and grey bars are grouped and simply called misclassified with no distinction of the level/degree with which they were misclassified.

**Figure 16:**
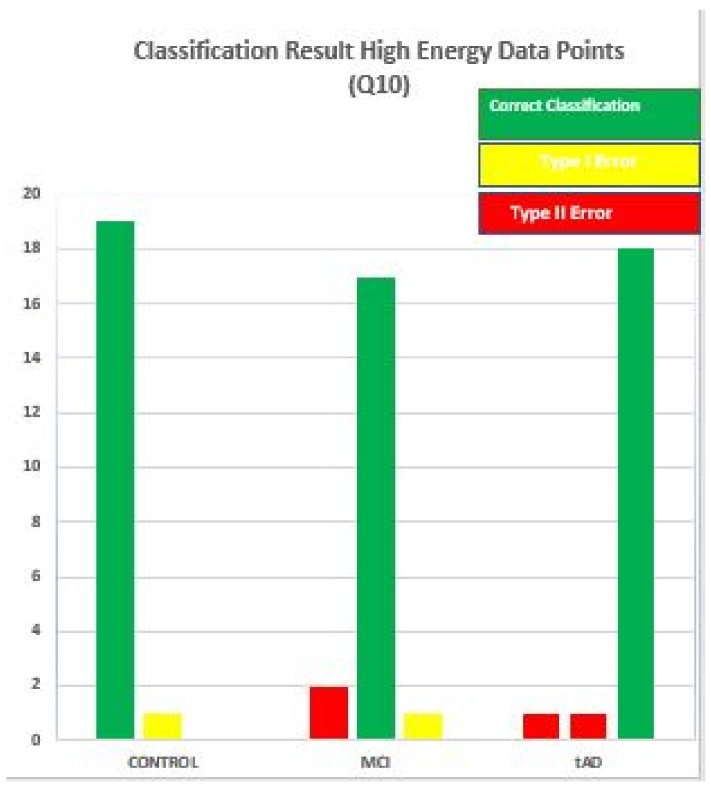
Bar chart using MDC-Q10-High classification to show error types associated with the classification of data points in relation to the disease stage they belong to.

See Figure 16 for a more descriptive representation of this scenario using MDC-Q10-high result as an example. With this chart it easier to identify the degree with which each data point wrongly classified was misclassified. In [16] and using Figure 16, two extreme scenarios were developed; one, it is impossible to commit type II error if a data point under consideration belongs to the CON stage. Secondly, only type I error can be committed with a TAD data point, but on the other hand, both error types can be committed if any of the distance algorithms is given an MCI data point to classify. Important issues to note with this discovery is that these extreme positions is always present. Similarly, the errors are inherent in the predisposed and non disposed strata.

A fuzzy-like criterion that takes these issues into cognizance was developed in [16]. The formulation assumes that each input data point is an MCI element and then classify such a data point as *incipient* TAD or relapsing to CON based on the outcomes of EE-KNN or MDC convolution methodology. The prediction with it is highly remarkable with over 98% of correctly classified instances.

## 4. Discussion and Conclusions

By matrix algebra and other operations with matrices, one can determine the magnitude and other properties of matrices. In cases where a matrix is a collection of tens of independent objects/observations or cases with overly skewed (or non-singular) matrices, the capabilities of matrix algebra can no longer be employed. One cannot use them to infer stratified classification as it becomes crucial to find other ways of validating the vectors of a matrix or categorize matrices based on the consensus behavior of its inherent vectors. Thus, quest for other methods such as computational frameworks presented in this report. The outcome of these methods demonstrates the benefits of advanced matrix convolutions with regards to harnessing high-throughput data as well as the diagnosis of Alzheimer’s disease.

In this report, we presented the limitation of Frobenius distance values evaluated ‘globally’ between matrix data points with regards to inferring classifications. The problem was reduced to a 2-dimensional problem, the methodology included redefining data points as a collection of vectors and a systematic approach was used to mine them using exponential euclidean distance functions.

Hitherto, to diagnose Alzheimer’s disease patients are meant to go through both mental and physical tests. The mental test may adopt one of the well known follow-up procedures such as clock drawing test (CDT), six item cognitive impairment test (SICIT), or the mini mental state examination (MMSE). The procedures are prone to individual clinician bias, longitudinal, and not dependent on protein biomarkers. Furthermore, they are usually inconclusive and not generalize-able.

On the other hand, the methodologies under review are novel and yields instant, accurate, dependable and generalize-able results. This was proven with the summarized result of [16]. It did not only classify/diagnose Alzheimer’s disease using MS saliva data, it went further to provide inputs to establish the degree of safety or probability with which a test data resides within the stage it belongs to. In addition to this is the PSS used; saliva samples are easy to assess or obtain regularly from participants.

As indicated above, the worst classification result of the methods under review is 20% better than default diagnosis. In many ramifications, default diagnosis is similar to the diagnoses procedures currently practiced today. This combined with its ability to produce instant results puts computational methods for the convolution of matrix data points as the state of the art tool for mining such data structures and diagnosing AD.

However, further studies are required to confidently bridge the gap and provide answers to questions related to Mass Spectrometry data acquisition techniques and dependable and generally available data banks. This report presented the review of works on the diagnoses of Alzheimer’s disease by mining MS-SELDI saliva data. The methodologies are easily extendable to other PSS and other diseases such as cancer. It provides the much needed platform for instant AD diagnoses, which provides the resources for medical practitioners to reach reliable decisions in timely manner.

## Figures and Tables

**Table 1:** Complete classification results with EE-KNN model.

Energy Level	Low	High
**ProteinChips**		
CM10	53%	80%
IMAC30	54%	82%
Q10	56%	85%

**Table 2:** Complete classification results with MDC model.

Energy Level	Low	High
**ProteinChips**		
CM10	55%	88%
IMAC30	55%	87%
Q10	60%	90%

**Table 3:** Classification results of default diagnosis.

Predisposed	NO	YES
NO	10	10
YES	20	20

**Table 4:** Classification results of the EE-KNN-CM10-Low Diagnosis.

Predisposed	NO	YES
NO	13	7
YES	8	32

## References

[1] Merchant M., Weinberger S.R. (2000). Recent advancement in surface-enhanced laser desorption/ionization-time of flight-mass spectrometry. Electrophoresis.

[2] Diamandis E.P. (2004). Mass spectrometry as a diagnostic and a cancer biomarker discovery tool opportunities and potential limitations. Mol. Cell. Proteom..

[3] Chapman J.D., Goodlett D.R., Masselon C.D. (2014). Multiplexed and data-independent tandem mass spectrometery for global proteome profiling. Mass Spectrom. Rev..

[4] Eng J.K., McCormack A.L., Yates J.R. (1994). An approach to correlate tandem mass spectral data of peptides with amino acid sequences in a protein database. J. Am. Soc. Mass Spectrom..

[5] Eng J.K., Fischer B., Grossmann J., MacCoss M.J. (2008). A fast SEQUEST cross correlation algorithm. J. Proteome Res..

[6] Perkins D.N., Pappin D.J., Creasy D.M., Cottrell J.S. (1999). Probability-based protein identification serching sequence databases using mass spectrometry data. Electrophoresis.

[7] Yadav A.K., Kumar D., Dash D. (2011). MassWiz: A novel scoring algorithm with target-decoy based analysis pipeline for tandem mass spectrometry. J. Proteome Res..

[8] Moore R.E., Young M.K., Lee T.D. (2002). An algorithm for evaluating sequest database search results. J. Am. Soc. Mass Spectrom..

[9] Keller A., Nesvizhskii A.I., Kolker E., Aebersold R. (2002). Empirical statistical model to estimate the accuracy of peptide identification made by MS/MS and database search. Anal. Chem..

[10] Nesvizhskii A.I., Keller A., Kolker E., Aebersold R. (2003). A statistical model for identifying proteins by tandem mass spectrometry. Anal. Chem..

[11] Cruz-Marcelo A., Guerra R., Vannucci M., Li Y., Lau C.C., Man T.K. (2008). Comparison of algorithms for pre-processing of SELDI-TOF mass spectromtry data. Bioinformatics.

[12] Wilhelm M., Schlegl J., Hahne H., Gholami A.M., Lieberenz M., Savitski M.M., Ziegler E., Butzmann L., Gessulat S., Marx H. (2014). Mass-spectromery-based draft of the human proteome. Nature.

[13] Röst H.L., Rosenberger G., Navarro P., Gillet L., Miladinović S.M., Schubert O.T., Wolski W., Collins B.C., Malmström J., Malmström L. (2014). OpenSWATH enables automated, targeted analysis of data-independent acquisition MS data. Nat. Biotechnol..

[14] Anyaiwe D.E., Wilson G.D., Geddes T.J., Singh G.B. (2018). Harnessing mass spectra data using KNN principle: diagnosing Alzheimer’s disease. ACM SIGBioinform. Rec..

[15] Anyaiwe O.E.D., Singh G.B., Wilson G.D., Geddes T.J. Weighted Manhattan Distance Classifier; SELDI data for Alzheimer’s disease diagnosis. Proceedings of the IEEE Congress on Evolutionary Computation (CEC).

[16] Anyaiwe O.E., Singh G.B. Fuzzy Prediction of Incipient Alzheimer’s Disease cases from Mild Cognitive Impaired cases. Proceedings of the 8th ACM International Conference on Bioinformatics, Computational Biology, and Health Informatics.

[17] Anyaiwe D.E., George W.D., Singh G.B. Classification by Clustering: Saliva MS-SELDI Data for Alzheimer’s Disease Diagnosis. Proceedings of the 2017 International Conference on Sensing, Diagnostics, Prognostics, and Control (SDPC).

[18] SELDI TECHNOLOGY Proteinchip Arrays and Reagents. https://www.bio-rad.com/webroot/web/pdf/lsr/literature/Bulletin_5524.pdf.

[19] Masood M.A., Khan M.N.A. (2015). Clustering techniques in bioinformatics. Int. J. Modern Comput. Sci..

